# How Follow-Up Period in Prospective Cohort Studies Affects Relationship Between Baseline Serum 25(OH)D Concentration and Risk of Stroke and Major Cardiovascular Events

**DOI:** 10.3390/nu16213759

**Published:** 2024-11-01

**Authors:** William B. Grant, Barbara J. Boucher

**Affiliations:** 1 Sunlight, Nutrition and Health Research Center, 1745 Pacific Ave., Suite 504, San Francisco, CA 94109, USA; 2 The Blizard Institute, Barts and The London School of Medicine and Dentistry, Queen Mary University of London, London E1 2AT, UK; bboucher@doctors.org.uk

**Keywords:** cardiovascular disease, causality, follow-up period/time, heart failure, hemorrhagic, hypertension, ischemic, prospective cohort study, stroke, vitamin D

## Abstract

**Background/Objectives:** Prospective cohort studies are useful for studying how biomolecular status affects risk of adverse health outcomes. Less well known is that the longer the follow-up time, the lower the association (or “apparent effect”) due to “regression dilution”. Here, we evaluate how follow-up interval from baseline to “event” affects the relationship between baseline serum 25-hydroxyvitamin D [25(OH)D] concentration and the later incidence of stroke and major cardiovascular events (MACEs). **Methods:** Findings for the relative risk (RR) of stroke and MACEs with respect to serum 25(OH)D concentrations at baseline from prospective cohort studies were plotted against mean follow-up time. Fifteen studies from mainly European countries and the United States were used for stroke and nine studies for MACEs. Linear regression analyses were used to study data for follow-up periods of up to 10 years and for more than 10 years. **Results:** For stroke, the linear regression fit for 1–10 years is RR = 0.34 + (0.065 × follow-up [years]), *r* = 0.84, adjusted *r*^2^ = 0.67, *p* < 0.001. No significant variations in association were found for studies with follow-up periods of 10–20 years. For MACEs, the linear fit for 1–8.1 years is RR = 0.61 + (0.055 × follow-up [years]), *r* = 0.81, adjusted *r*^2^ = 0.59, *p* = 0.03. **Discussion:** The shorter the follow-up period, the greater the apparent effect of better vitamin D status in reducing risk of stroke and MACEs. In addition, the apparent effect of higher 25(OH)D concentration found for the shortest follow-up time is more than twice as great as the estimate based on average follow-up intervals for all studies. Mechanisms have been found to explain how higher serum 25(OH)D concentrations could reduce risk of stroke and MACEs. Randomized controlled trials have not shown that vitamin D supplementation significantly reduces risk of either stroke or MACEs, probably because risk of both outcomes increases rapidly below 15 ng/mL (38 nmol/L) and it is difficult in Western developed countries to enroll enough participants with concentrations that low. Nonetheless, vitamin D’s role in reducing risk of stroke and MACEs could be considered causal on the basis of an evaluation of the evidence using Hill’s criteria for causality in a biological system. **Conclusions:** Serum 25(OH)D concentrations above 20 ng/mL are associated with significantly reduced risk of stroke and MACEs prospectively and in an apparent causal manner. Raising serum 25(OH)D concentrations to >20 ng/mL should, therefore, be recommended for everyone likely to be at risk for stroke or MACEs and indeed in the general population.

## 1. Introduction

The prospective cohort study is a type of observational study commonly used to assess how dietary and lifestyle factors and biological variables affect health outcomes. Participants are recruited and enrolled, information relevant to the study is obtained from each participant, and participants are followed up for a period during which various health outcomes of interest are recorded. Afterward, health outcome rates are analyzed statistically in relation to data assessed at baseline during enrollment. Most such studies do not remeasure any variables assessed during the follow-up period. Therefore, an underestimation of risk associations due to “regression dilution” generally occurs in long-term follow-up in prospective studies, as outlined by Clarke and colleagues in 1999 [1], who reported repeated measurements over 25 years for systolic and diastolic blood pressure and blood cholesterol for participants in the Framingham Study: their findings showed that the range of associations from high to low for the first and fifth quantile shrank by 65%, 75%, and 57%, respectively. Although the article had 897 citations by 31 August 2024, according to Google Scholar, that finding seems not to have had much effect on the conduct of prospective cohort studies or, more importantly, on meta-analyses made using such studies though the concentration of 25-hydroxyvitamin D [25(OH)D] in serum changes over time. For example, a study in Norway reported that the correlation coefficient, *r*, for serum 25(OH)D concentrations measured in 2668 participants in 1994 and again in 2008 and adjusted for season of measurement was 0.42 [2], indicating low-to-moderate correlation. In comparison, the correlation coefficients for systolic blood pressure, diastolic blood pressure, pulse, body mass index (kilograms per square meter of body surface area), serum total cholesterol, and triglycerides between 1994 and 2008 were 0.47, 0.42, 0.46, 0.83, 0.37, and 0.49, respectively, again indicating only moderate correlation. The significant effect of follow-up period on the associations of serum 25(OH)D with health outcome has been known for cancer since 2011 [3] and for all-cause mortality rate since 2012 [4], yet that phenomenon was overlooked in a highly cited meta-analysis of risk of colorectal cancer with respect to serum 25(OH)D concentration in 2019 [5], as Muñoz and Grant pointed out in 2022 [6]. By ignoring this factor, the beneficial effects of higher serum 25(OH)D concentration are underestimated, making them less likely to be used to guide research or public health policies.

Recently, researchers showed that the same effect (of changes in vitamin D status over time reducing its associations with health outcomes) is found for the risk of cognitive impairment, dementia, and Alzheimer’s disease. A 2024 meta-analyses included 15 prospective studies regarding dementia and/or Alzheimer’s disease and 9 regarding cognitive impairment [7]. As shown in plots of risk ratio for low versus high 25(OH)D concentration with different follow-up periods, linear decreases emerged in the regression fit to the data from near 2.0 for the shortest follow-up periods (near 4–5 years) to near 1.0 for follow-up periods near 13 years [8].

Stroke is an important cause of disability and death. In 2016, an estimated 13.7 million new incident strokes occurred globally, of which about 87% were ischemic strokes [9]. In 2017, an estimated 1.12 million incident strokes occurred in the European Union, with 9.53 million stroke survivors and 7.06 million disability-adjusted life years lost because of stroke [10]. A prospective study of 418,329 participants in the European Prospective Investigation into Cancer and Nutrition (EPIC) included an analysis of dietary risk factors for stroke [11]. Risk of ischemic stroke was inversely associated with consumption of fruit and vegetables, dietary fiber, and dairy foods, whereas risk of hemorrhagic stroke was positively associated with egg consumption. In the USA in 2021, it was estimated that there were 932 thousand deaths from cardiovascular disease (CVD), 696 thousand from heart disease, and 163 thousand from stroke [12].

A 2021 meta-analysis of prospective cohort studies of risk of stroke with respect to serum 25(OH)D by Su and colleagues [13] suggested to us that a reanalysis of stroke and MACE data should be made allowing for intervals from baseline to event, as in a recent meta-analysis of relevance by Xiong and colleagues [14]. There was also the meta-analysis of incidence of major cardiovascular events (MACEs) by Zhang and colleagues [15], suggesting that a similar analysis of the effect of follow-up time on the incidence of MACEs be conducted. The goal of this review, therefore, is to assess how follow-up period affects the associations found between baseline serum 25(OH)D concentrations and incidence rates of stroke and MACEs.

## 2. Materials and Methods

The data used here are from the prospective cohort studies examined by both Su and colleagues [13] and Xiong and colleagues [14]. Table 1 and Table 2 list the studies in ascending order of follow-up interval. Table 2 includes the adjusted odds ratio/relative risk (OR/RR) for each study reported by Su and colleagues [13] and by Xiong and colleagues [14] and is verified by inspection of the studies, and it gives the follow-up period and 25(OH)D concentration association [correlation rate] reported in each article. Each study listed the variables included in adjusting the OR/RR. For example, Anderson and colleagues [16] included hypertension, hyperlipidemia, diabetes mellitus (DM), and peripheral vascular disease. Judd and colleagues [17] adjusted for socio-demographic factors, co-morbidities, and laboratory values including parathyroid hormone.

We excluded four studies from the analysis: two did not have enough information on follow-up period or on how 25(OH)D concentrations were compared, and two because they were based on dietary vitamin D intake.

Table 3, Table 4 and Table 5 list information from eight studies used to investigate the effect of follow-up period on risk of incidence of MACEs. The tables do not include studies regarding cardiovascular disease (CVD) mortality rate because mortality can be significantly affected by treatment, thereby obscuring the effect of serum 25(OH)D concentration. MACE incidence can also be lowered through intervention. For example, lowering blood pressure pharmacologically by 5 mmHg can lower incidence of MACEs by 5–15% depending on baseline blood pressure [35].

Data were analyzed using SigmaStat 4.0 (Grafiti, Palo Alto, CA, USA). Data plots were made using KaleidaGraph 4.5.4 (Synergy Software, Reading, PA, USA).

## 3. Results

**Figure 1:**
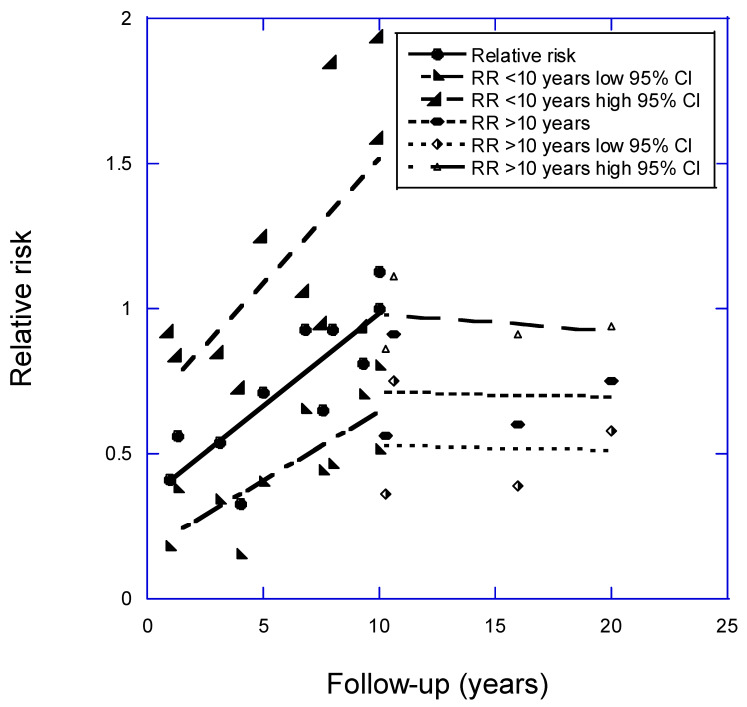
Plot of relative risk for stroke versus years of follow-up with respect to high vs. low 25(OH)D concentration, with regression fits to studies of less than 10 years and for those carried out over more than 10 years; 95% CI, 95% confidence interval. Equation for regression fit to RR for follow-up period < 10 years is RR = 0.34 + (0.065 × follow-up [years]), *r* = 0.84, adjusted *r*^2^ = 0.67, *p* < 0.001.

Figure 1 shows a plot of the data from Table 1. The data were analyzed after division into two groups of 1–10-year or >10-year follow-up periods between baseline vitamin D and determination of the incident events. The determination of the dividing year was made by inspecting the data plot. The graph shows a good linear fit to the data for follow-up periods of 1–10 years: RR = 0.34 + (0.065 × follow-up [years]), *r* = 0.84, adjusted *r*^2^ = 0.67, *p* < 0.001. The regression coefficients for the data were reported for the shortest interval to events post-baseline, one year, =−0.41 (95% CI, 0.22–0.75), but regression coefficients for the data on follow-up intervals >10 years showed no significant change with increasing follow-up interval. In addition, the 95% confidence interval (95% CI) values are smaller for the regression coefficients for the shorter follow-up intervals, though this finding may simply reflect the higher events rates in the studies with shorter follow-up periods. Although most studies gave results for all strokes (hemorrhagic and ischemic), ischemic strokes are much more frequent than hemorrhagic strokes in the countries studied [9]. Some reports included in the present study, however, showed that hemorrhagic strokes had weaker associations with baseline serum 25(OH)D concentrations than did ischemic stokes [17,28].

**Figure 2:**
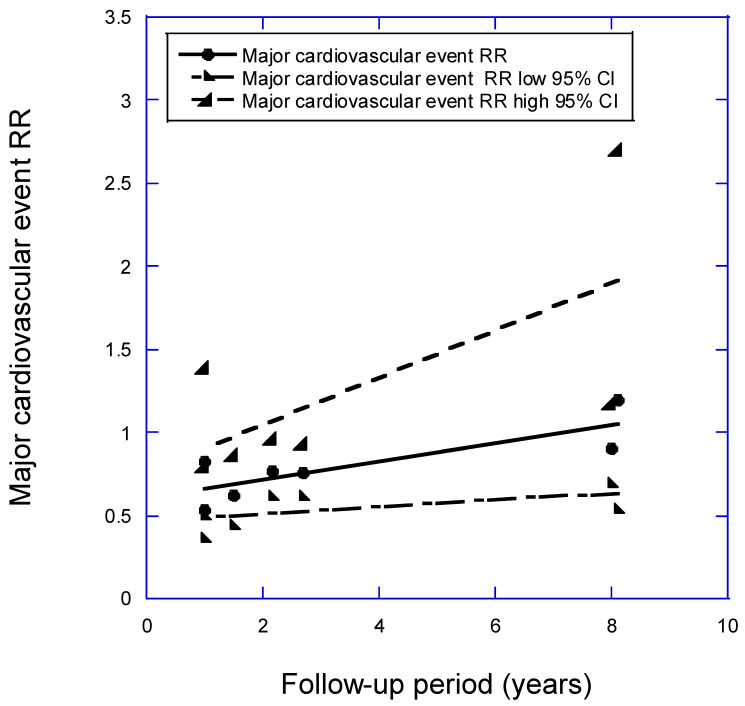
Plot of relative risk of a major cardiovascular event (MACE) versus mean follow-up period for high versus low 25(OH)D concentration. Equation for regression fitted to RRs over follow-up periods <10 years is RR = 0.61 + (0.055 × follow-up [years]), *r* = 0.81, adjusted *r*^2^ = 0.59, *p* = 0.03.

Figure 2 shows a plot of the data from Table 2 for MACE vs. follow-up period. The equation for the regression fit to the RR for high versus low 25(OH)D concentration for a follow-up period <10 years is RR = 0.61 + (0.055 × follow-up [years]), *r* = 0.81, adjusted *r*^2^ = 0.59, *p* = 0.03. The regression fit to the data for the shortest follow-up period, one year, is RR = 0.66 (95% CI, 0.48–0.91). This is a lower reduction than that found for stroke, which for one year for low versus high baseline 25(OH)D concentration is RR = 0.41 (95% CI, 0.22–0.75).

## 4. Discussion

This study found that mean follow-up period significantly affects the determination of relative risk for stroke and MACEs in relation to 25(OH)D concentration measured at time of enrollment. The effect was much stronger for stroke incidence than for MACEs. A search for “stroke” in the MACE studies used in this analysis found that the four longest follow-up studies did not include stroke, while three of the four reporting MACE rates per se mention stroke. Only Beska 2019 [37] mentioned stroke, noting that very few stroke events were observed. Thus, it seems that the MACE findings are independent of stroke. The tentative conclusion is that the risk of stroke for low serum 25(OH)D concentration is much stronger than for other CVD events.

The meta-analysis of RRs calculated for stroke rates in relation to baseline serum 25(OH)D concentration, based on 21 prospective observational studies by Su and colleagues [13], reported an average value of 0.78 (95% CI, 0.70–0.86), whereas that by Xiong and colleagues [14], based on 21 slightly different prospective observational studies, showed an average value of 0.78 (95% CI, 0.70–0.87). Those values are approximately half the reduction seen for the 1-year follow-up period and 44% of the regression found for a “zero” follow-up period in the present study. That finding offers more evidence to support the suggestion that failure to consider the effect of follow-up period in meta-analyses of observational studies with long follow-up times can lead to considerable underestimates of the effect of the baseline variable studied. As a result, public policy recommendations are likely to be less strong than they should be.

In a 2020 article, Shi and colleagues [42] calculated the dose–response relationship for 25(OH)D concentration and stroke risk by using mostly the same observational studies as Su and colleagues [13]. The result, shown in Figure 2 in Ref. [42], was that risk decreased by ~20% with increasing vitamin D status between zero and ~20 ng/mL, with no further risk reduction between 20 and 40 ng/mL. However, as a result of not having accounted for variation in follow-up period, that analysis underestimates the reduction actually achievable with better vitamin D status. By contrast, the study does show that the main risk reduction occurs with increases in serum 25(OH)D up to 15 ng/mL. Very few randomized controlled trials (RCTs) could enroll participants with mean 25(OH)D concentrations that low, for ethical reasons, unless conducted in a country where such low 25(OH)D concentrations commonly persist. For example, many Middle Eastern countries have diets low in animal products, and their peoples often wear clothing that covers most of the body and stay indoors during the hot summers [43]. Results of a stratified randomized field trial of vitamin D supplementation in pregnant women in Iran reported that the mean baseline 25(OH)D concentration was 11 ng/mL [44]. By supplementing the women at one hospital with enough vitamin D to raise 25(OH)D concentrations to above 20 ng/mL, significant reductions occurred for gestational diabetes, pre-eclampsia, and preterm birth in comparison with outcomes in a comparable hospital where pregnant women were not supplemented.

Two factors might help explain why the results for MACEs with respect to 25(OH)D concentration were weaker than those for stroke: since mortality was an important component of MACEs in several of the studies, the participants were very likely to be treated in a variety of ways, thereby reducing the impact of 25(OH)D concentration; also, the individual MACE components may have different 25(OH)D concentration–health outcome relationships.

Comparing the differences in outcomes for stroke, CVD, and cancer is insightful. Cancer incidence and mortality rates used to have very large geographic variations in the United States [6], but stroke and CVD have not shown similar geographical variations. However, they do have more pronounced seasonal variations in mortality rates than cancer, with 20% higher mortality rates in winter than in summer in northern hemisphere countries [45]. The seasonal variation is due in part to the seasonal variations in serum 25(OH)D concentrations related to solar UVB doses [46,47]. Cancers have large geographical variations in midlatitude counties such as the United States [48] because serum 25(OH)D concentrations greater than 50 ng/mL reduce risk in comparison with lower concentrations, as shown for colorectal cancer [49] and breast cancer [50]. The differences in the 25(OH)D concentration–risk relationship also explain why the VITAL study—with a mean 25(OH)D concentration of 31 ng/mL for the participants in the vitamin D treatment arm whose serum 25(OH)D was measured—showed significantly reduced all-cancer incidence rates for participants with BMI < 25 kg/m^2^ and reduced all-cancer mortality rates for the entire treatment group, but no significant effects for cardiovascular disease [51]. In that RCT, MACEs occurred in 3.1% of the vitamin D treatment arm and in 3.2% of the placebo arm, whereas strokes occurred in 1.1% of the vitamin D treatment arm and 1.2% of the placebo arm.

An important question is how rapidly vitamin D might reduce risk of adverse brain and other health outcomes. As discussed in the analysis of follow-up period for cognitive function, one RCT has shown significant beneficial effects in improving cognitive function during 1 year of vitamin D supplementation [52]. To examine that question further, we searched Google Scholar for representative RCTs that reported a beneficial effect on brain health in less than 1 year (Table 6), and significant benefits were found for depression and cognitive function within one year; those studies also showed that raising serum 25(OH)D concentrations can lead to significant improvements in brain health within a year. Three of those papers dealt with studies from China, India, and/or Iran, where serum 25(OH)D concentrations are generally low, a situation that means that RCTs in those communities are more likely to be able to show health benefits from supplementation than studies conducted in countries where their populations have much higher mean 25(OH)D concentrations.

For many reasons, serum 25(OH)D concentrations change over time scales from months to years (Table 7).

Several mechanisms have been identified that help explain how higher 25(OH)D concentrations can reduce the risk of stroke. For example, a 2014 review [68] stated that vitamin D influences neuronal function by binding to vitamin D receptors that can act as transcription factors and regulate gene expression [69]. Particularly in the nervous system, vitamin D’s biological effects appear to arise from stimulation of neurotrophic factors, quenching of oxidative hyperactivity, and regulation of autoimmune responses [70]. Increasing 25(OH)D concentrations through vitamin D supplementation causes more genes to be expressed (or, more rarely, fewer). Thirty healthy adults were randomized to receive 600, 4000, or 10,000 IU/day of vitamin D_3_ for 6 months. The study showed a dose-dependent 25(OH)D alteration in broad gene expression with 162, 320, and 1289 genes, respectively, upregulated or downregulated in white blood cells [71].

Evidence also exists that vitamin D supplementation in vitamin D-deficient subjects can reduce serum concentrations of matrix metalloproteinases MMP-2 and MMP-9, as well as of their inhibitor, TIMP-1, and of C-reactive protein [72]. That finding is relevant because people who develop MACEs have raised MMP-9 concentrations, and MMP-9 is an important risk factor for vulnerable atherosclerotic plaque [73].

Hypertension is a risk factor for stroke. A dose–response meta-analysis showed that higher adherence to antihypertension medications reduced the risk of hemorrhagic stroke by 45% (RR = 0.55 [95% CI, 0.42–0.72]) and of ischemic stroke by 26% (RR = 0.74 [95% CI, 0.69–0.79]) [74]. A meta-analysis of serum 25(OH)D concentration on risk of hypertension in the general population based on cohort studies reported a significant increase of 38% (RR = 1.38 [95% CI = 1.14–1.64]) as 25(OH)D concentrations decreased from 75 to 15 nmol/L [75]. The same paper showed no effect of vitamin D supplementation on systolic or diastolic blood pressure on the basis of 27 studies. However, three supplementation studies reported significant or near-significant reductions in systolic blood pressure [76,77,78]. Also, an open-label study in Canada in which participants took enough vitamin D_3_ to increase serum 25(OH)D concentrations above 40 ng/mL reported significant reductions in the prevalence of hypertension [79]. Thus, there is some evidence that does show that vitamin D repletion can lower blood pressure and reduce the risk of established hypertension.

Table 8 lists more mechanisms by which vitamin D can reduce risk of stroke and MACEs.

RCTs have not supported the role of better vitamin D provision in reducing risk of stroke. A 2020 systematic review and meta-analysis of vitamin D supplementation and incidence of stroke included 13 RCTs [90]. The mean age was 66 years, and the mean follow-up time was 3.1 years. The mean baseline 25(OH)D concentration for studies that reported values was 19.4 ng/mL (range, 8.8–25.4 ng/mL). The percentage of participants in those 13 trials who experienced a stroke was 2.1% in both the treatment and control arms, resulting in an RR for stroke of 1.00 (95% CI, 0.91–1.10). Inspecting the baseline characteristics of participants in those trials (in Table 1 in Nudy 2021 [90]) shows that participants were being studied for various adverse health effects, including arthritis index pain, asthma exacerbations, progression to type 2 DM, falls and fractures, insulin sensitivity, and renal function. In other words, none of the trials was established specifically to evaluate the role of vitamin D supplementation, in deficiency, on the risk of stroke incidence.

A 2024 review included a different group of five vitamin D RCTs to assess the risk of ischemic strokes [91]. Mean baseline 25(OH)D concentrations were from 66 ± 23 to 77 ± 25 ng/mL in four trials and 38 ± 16 ng/mL in one trial. Follow-up duration ranged from 3.3 to 5.3 years. All those trials had CVD outcome as a primary outcome. Again, no significant difference in stroke risk was found between the vitamin D treatment and control arms, as could have been predicted from the baseline vitamin D status.

Even if the trials had been set up to test for stroke incidence, they probably would not have shown a beneficial effect for several reasons. First, nearly all vitamin D RCTs have been based on guidelines for pharmaceutical drugs. In such trials, the control arm does not receive the drug. That is not the case for vitamin D trials because vitamin D is a naturally occurring substance required for life and supplement use is often permitted in controls and it is considered unethical not to give the control group a minimal vitamin D_3_ supplement [92]. In 2014, Heaney outlined guidelines for trials of nutrient provision [93].

The main steps appropriate for vitamin D [94] are as follows:

1. Measure 25(OH)D concentrations and include participants with low concentrations appropriate for the outcome of interest.
2. Give a vitamin D dose large enough to raise 25(OH)D concentrations to levels at which beneficial effects are normally observed.
3. Measure achieved 25(OH)D concentrations and adjust vitamin D dosage to maintain adequate achieved levels.
4. Analyze results with respect to achieved vitamin D concentrations.

Another major reason for failure of vitamin D RCTs is the enrollment of people with relatively high 25(OH)D concentrations. Other reasons include giving relatively low doses, i.e., too small to correct deficiency, and permitting participants in the control and sometimes also the treatment arms to continue taking moderate vitamin D supplements. Analyzing results by intention to treat rather than by initial and achieved vitamin D status is another common mistake in vitamin D RCT analysis. Those common failures have been discussed in two 2022 reviews [95,96].

Risk rates for the incidence of stroke and other CVD events increase rapidly as serum 25(OH)D concentrations fall below 20 ng/mL [23,97]. Thus, for example, the large VITAL study [51], which enrolled participants whose mean baseline 25(OH)D concentration was 31 ng/mL in the vitamin D treatment arm, had no chance of finding any significant reduction in CVD from supplementation at 2000 IU/day of vitamin D_3,_ The failure to recruit participants with deficiency and permitting those in the placebo arm to take up to 600 or, for those over 70 years, 800 IU/day of vitamin D, also reduced the possibility of any significant findings being able to emerge from the VITAL trial.

A stratified randomized field trial of vitamin D supplementation for pregnant women in Iran [44] shows how to design and carry out any trial of vitamin D RCT more appropriately [though in that instance for pregnancy complications rather than for stroke]. Firstly, participants had mean 25(OH)D concentrations of about 11 ng/mL. Second, serum 25(OH)D concentrations for the vitamin D treatment arm were measured during the trial and used to adjust vitamin D doses so as to ensure that 25(OH)D concentrations greater than 20 ng/mL were achieved and maintained throughout the trial, as confirmed by follow-up 25(OH)D values, while participants in the control arm received no vitamin D supplements. Outcomes for gestational diabetes, pre-eclampsia, and preterm delivery were then evaluated with respect to achieved 25(OH)D concentrations, and highly significant reductions in those outcomes were found. Adverse pregnancy outcomes, including pre-eclampsia, gestational diabetes mellitus, and preterm delivery, were decreased by 60% (OR = 0.4 [95% CI, 0.3–0.6]), 50% (OR = 0.5 [95% CI, 0.3–0.9]), and 40% (OR = 0.6 [95% CI, 0.4–0.8]), respectively, in the screening site. Those findings were in contrast with results from the standard vitamin D RCTs for pregnant women that were used to prepare a recent Cochrane review [98]. It reported, “The evidence is very uncertain about the effect of supplementation with vitamin D during pregnancy compared to placebo or no intervention on pre-eclampsia (risk ratio (RR) 0.53, 95% confidence interval (CI) 0.21 to 1.33; 1 study, 165 women), gestational diabetes (RR 0.53, 95% CI 0.03 to 8.28; 1 study, 165 women), preterm birth (<37 weeks) (RR 0.76, 95% CI 0.25 to 2.33; 3 studies, 1368 women)”.

The mainstream medical system relies on RCTs to show effectiveness and to ensure limited adverse effects before approving pharmaceutical drugs for general use, but, as discussed, vitamin D RCTs based on guidelines for pharmaceutical drugs are not appropriate. In addition, Hill in 1965 proposed another way to examine causality in a biological system [99]. The criteria appropriate for vitamin D included strength of association, consistent findings in different populations, temporality, biological gradient, plausibility (e.g., mechanisms known), coherence with known science of the day, experiment, and analogy. Necessary adjustment for confounding factors and bias were added later [100]. A 2024 review discussed how those considerations have influenced epidemiologic methods [101]. For vitamin D, examination of Hill’s criteria for causality of inadequate vitamin D provision for various health outcomes (diseases) [96], including cardiovascular disease [102], suggested that the criteria were generally satisfied. Ironically, the main limitation to using Hill’s criteria of causation was in finding reliable evidence from experimentation owing to the problems with RCTs, as discussed above and as recently reviewed [87].

It is notable that when results from RCTs are not available, observational studies have been used to evaluate and establish causal relationships. This was performed, for example, for smoking as a primary cause of lung cancer by Doll and Hill [103,104]. Table 9 uses Hill’s criteria to briefly outline the evidence that vitamin D reduces risk of stroke. While most of the criteria appear to be satisfied, experimental verification has not been satisfied. Again, the most likely reason is that RCTs have not found that vitamin D reduces risk of stroke or MACEs, again because they have been based on guidelines for pharmaceutical drugs rather than nutrients [93,95,96]; in addition, no RCTs have enrolled sufficient numbers of participants low 25(OH)D concentrations, i.e., at less than 15 ng/mL. As Hill noted, not all criteria have to be satisfied for causality to be likely, though the more that are, the stronger the claim [99].

### Strengths and Weaknesses

Strengths include using several cohort studies in analysis and analyzing the meta-analysis results in terms of the follow-up periods used.

One limitation is that the analyses are based on observational studies. Another is that the findings relating vitamin D status to stroke risks are not supported by RCTs. A third is that nearly all of the studies are from Western developed countries. As a result, they may not be applicable to developing countries. Also, most CVD events are in part related to Western dietary patterns, although this has not been investigated in this work.

## 5. Conclusions

From our findings, the risks of stroke and of acute cardiovascular events are likely to be minimized by ensuring population serum 25(OH)D concentrations are maintained at or above 20–30 ng/mL. In normal-weight people, that level can be achieved with supplementation of at least 1000 IU/day of vitamin D_3_, though 2000 IU/day would be more reliable [107]. Obese people, however, should take 2–3 times higher doses, and overweight individuals should take 1.5 times as much [108].

## Figures and Tables

**Table 1:** Baseline data for stroke studies listed in Su and colleagues [13] and Xiong and colleagues [14] used in this study and listed by increasing follow-up period.

Country	Patient Characteristics	Mean Age (± SD) or Range (yrs)	BMI (± SD) (kg/m^2^)	F, M (%)	Stroke Type	*N_S_*	*N_C_*	Ref.
Germany	Left ventricular assist device implants	62 (37–81)	23 ± 3	0, 100	All	25		Zittermann 2016 [18]
		57 (49–66)	26 ± 5	15, 85			129	
USA	Community hospital	55 ± 21	NA	75, 25	All	208	25,818	Anderson 2010 [16]
USA	B and W community dwellers	65	65	55, 45	I	536	1069	Judd 2016 [17]
Germany	Diabetic and on hemodialysis	66 ± 8	27 ± 5	40, 60	All	89	1019	Drechsler 2010 [19]
New Zealand	Healthy community dwelling	74 ± 4	NA	100, 0	All	59	1412	Bolland 2010 [20]
Germany	Population-based	65% 50–65; 35% 65–74	27 ± 5	59, 41	All	353	7356	Perna 2013 [21]
Germany	Population-based	51	NA	58, 42	All	471	1661	Kuhn 2013 [22]
USA	Stable CVD	66 ± 11	29	19, 81	All	49	897	Welles 2014 [23]
Denmark	General population	58 (48–68)	26 ± 3	52, 48	I	960	~115,000	Afzal 2017 [24]
Finland	Population-based	65–99	NA	52, 48	All	70	685	Marniemi 2005 [25]
Denmark	General population	49 (41–73)	26	50, 50	All	316	8830	Skaaby 2013 [26]
Hong Kong	Osteoporosis study, Chinese	63 ± 10	NA	63, 37	All	244	3214	Leung 2017 [27]
					I	205	3253	
Netherlands	Population-based	65 ± 10	27 ± 4	57, 43	All	735	8603	Berghout 2019 [28]
Denmark	Osteoporosis study	50 ± 2	25 ± 5	100, 0	All	89	1924	Schierbeck 2012 [29]
USA	Population-based	57	NA	57, 43	All	804	11,354	Schneider 2015 [30]

Data for participants with incident stroke: B, Black; BMI, body mass index (kilograms per square meter of body surface area); CVD, cardiovascular disease; F, female; I, ischemic; M, male; NA, not available; *N_C_*, number of controls; *N_S_*, number with incident stroke; SE, standard error; W, white.

**Table 2:** Findings for stroke studies from data for stroke studies listed in Su and colleagues [13] and Xiong and colleagues [14]. They are listed in order of increasing follow-up period.

Type of Stroke	25(OH)D Comparison (ng/mL)	Follow-Up (yrs)	Inc or Mor	Adjusted OR/RR (95% CI)	Ref.
		NA		1.41 (0.64–3.13)	Guo 2017 * [31]
		NA		1.19 (0.79–1.79)	Leu Agelii 2017 * [32]
All	≥10 vs. <10	1	Inc	0.42 (0.14–1.28)	Zittermann 2016 [18]
All	>30 vs. ≤15	1.3	Inc	0.56 (0.38–0.84)	Anderson 2010 [16]
All	>30 vs. <20	3.1	Inc	0.54 (0.34–0.85)	Judd 2016 [17]
All	>30 vs. ≤10	4	Inc	0.33 (0.15–0.73)	Drechsler 2010 [19]
All	≥20 vs. <20	5	Inc	0.71 (0.40–1.25)	Bolland 2010 [20]
	per + 10 *	6.8	Inc + mor	0.91 (0.81–1.02)	Perna 2013 [21]
All	<12 vs. ≥20			0.76 (0.55–1.05)	
All	Q4 (27 median) vs. Q1 (12 median) *	7.6	Inc	0.60 (0.59–1.09)	Kuhn 2013 [22]
	≥20 vs. <10			0.65 (0.44–0.95)	
All	≥20 vs. <20	8.0	Inc	0.93 (0.46–1.85)	Welles 2014 [23]
I	≥20 vs. <10	9.3	Inc	0.81 (0.70–0.94)	Afzal 2017 [24]
All	High vs. low tertile	10	Inc + mor	1.00 (0.51–1.94)	Marniemi 2005 [25]
All	Middle vs. low tertile			0.88 (0.49–1.61)	
	Fourth vs. first quartile	10	Inc	1.13 (0.80–1.59)	Skaaby 2013 [26]
All	Lowest vs. highest quintile	10.3	Inc	0.56 (0.36–0.86)	Leung 2017 [27]
I	Middle vs. highest quintile			0.55 (0.35–0.86)	
All	One 25(OH)D SD increase	10.6	Inc	0.91 (0.75–1.11)	Berghout 2019 [28]
All	≥20 vs. <20	16	Inc or mor	0.60 (0.39–0.91)	Schierbeck 2012 [29]
All	>440 vs. <110 IU/day vitamin D	19.3	Mor	0.66 (0.49–0.89)	Sheerah 2018 * [33]
All	≥31 vs. <17	20	Inc	0.75 (0.58–0.94)	Schneider 2015 [30]
All	>4 vs. <1.1 µg/day	34	Inc	0.82 (0.68–0.99)	Kojima 2012 * [34]

*, omitted from the analysis; 25(OH)D, 25-hydroxyvitamin D; 95% CI, 95% confidence interval; I, ischemic; Inc, incidence; mor, mortality; NA, not available; OR, odds ratio; RR, relative risk.

**Table 3:** Participant information for MACEs in prospective cohort studies from studies in Zhang and colleagues [15] used in this study, listed in order of increasing follow-up period.

Country	Mean Age (±SD) (yrs)	BMI (±SD) (kg/m^2^)	F, M (%)	*N_MCDE_*	*N_C_*	Ref.
Italy	67 ± 12	27 ± 4	28, 72	125	689	de Metrio 2015 [36]
UK	81 ± 5	27 ± 5	38, 62	76	224	Beska 2019 [37]
UK	66 ± 13	NA	28, 72	224	1035	Ng 2013 [38]
Italy	67 ± 12	27 ± 4	29, 17			Aleksova 2020 [39]
Italy	68 ± 11	28 ± 5	27, 73	174	531	Verdoia 2021 [40]
USA	66 ± 11	29	19, 81	49	897	Welles 2014 [23]
Germany	>60	28	27, 73	148	977	Grandi 2010 [41]

BMI, body mass index (kilograms per square meter of body surface area); F, female; M, male; NA, not available; *N_MCDE_,* number with a major cardiovascular disease event; *N_C_*, number of controls.

**Table 4:** Patient characteristics and type of MACE for studies in Zhang and colleagues [15] used in this study, listed in order of increasing follow-up period.

Patient Characteristics	Type of Events	Ref.
ACS	Death, major bleeding, acute pulmonary edema, cardiogenic shock, significant tachyarrhythmias, acute kidney injury	de Metrio 2015 [36]
After non-ST elevation ACS	Death, acute coronary syndrome, unplanned repeat revascularization, significant bleeding, or stroke	Beska 2019 [37]
Acute MI	Death, HF, angina/MI	Ng 2013 [38]
Survivors of MI	Death, angina/MI, and heart failure	Aleksova 2020 [39]
CAD undergoing percutaneous coronaryintervention	Death, MI, target vessel revascularization	Verdoia 2021 [40]
Stable CVD	Cardiovascular events (HF, MI, stroke, or cardiovascular death)	Welles 2014 [23]
Stable CHD	Cardiovascular event incidence (fatal and nonfatal, including MI, stroke, and death due to cardiovascular diseases) and death	Grandi 2010 [41]

CAD, coronary artery disease; CHD, coronary heart disease; CVD, cardiovascular disease; HF, heart failure; MACE, major cardiovascular event; MI, myocardial infarction.

**Table 5:** Findings for MACE rates in prospective cohort studies in Zhang and colleagues [15], listed in order of increasing follow-up period.

25(OH)D Comparison (ng/mL)	Follow-Up (yrs)	Adjusted RR (95% CI) for High vs. Low 25(OH)D	Reference
>9 vs. <9	1.0	0.54 (0.36–0.80)	de Metrio 2015 [36]
>12 vs. <12	1.0	0.83 (0.50–1.39)	Beska 2019 [37]
>7.3 vs. <7.3	1.5	0.62 (0.44–0.87)	Ng 2013 [38]
>20 vs. <20	2.2	0.77 (0.61–0.96)	Aleksova 2020 [39]
≥21.6 vs. <21.6	2.7	0.76 (0.61–0.93)	Verdoia 2021 [40]
≥20 vs. <20	8.0	0.90 (0.69–1.18)	Welles 2014 [23]
Quartiles	8.1	1.20 (0.54–2.70)	Grandi 2010 [41]

25(OH)D, 25-hydroxyvitamin D; 95% CI, 95% confidence interval; MACE, major cardiovascular event; RR, relative risk.

**Table 6:** Results of short-term vitamin D supplementation on brain health.

Participants	Duration (wks)	Condition	Intervention	Outcomes	Ref.
Meta-analysis of nine clinical trials, China and Iran	8–52	Mental health	50,000 IU/wk or 2 wks or higher single dose	Beck Depression Inventory, weighted mean difference, −3.9 (95% CI, −5.2 to −2.7)	Jamilian 2019 [53]
A total of 46 patients, India; baseline 25(OH)D: N/A	12	Major depressive disorder	Usual treatment or usual treatment plus 3 million IU of vitamin D	Significantly greater improvement in depression score with vitamin D than placebo and also quality of life	Vellekkatt 2020 [54]
A total of 64 patients under methadone maintenance treatment, Iran; baseline 25(OH)D: 14 ± 4 ng/mL	24	Cognitive function	50,000 IU or placebo/2 wks	Vitamin D treatment resulted in significant improvement in Iowa Gambling Task, Verbal Fluency Test, Reverse Digit Span, and visual working memory	Ghaderi 2020 [55]
A total of 42 women, USA mean age 58 ± 6 years, BMI, 30.0 ± 3.5 kg/m^2^; baseline 25(OH)D: 23 ± 6 ng/mL	52	Cognitive outcome	600, 2000, or 4000 IU/day of vitamin D_3_	2000 IU/day group had improved visual and working memory and learning; the 4000 IU/day group had slower attention reaction time	Castle 2020 [56]

25(OH)D, 25-hydroxyvitamin D; 95% CI, 95% confidence interval.

**Table 7:** Why 25(OH)D concentrations may change over time.

Reason	Ref.
*For increases in 25(OH)D concentrations*	
Increased awareness of overall benefits of vitamin D	Rooney 2017 [57]
Increase amount of omega-3 fatty acid supplementation	Alhabeeb 2022 [58]
Increased vitamin D supplementation after menopause	Perez-Lopez 2020 [59]
Retire from work	Aspell 2019 [60]
*For variable changes in 25(OH)D concentrations*	
Change geographic location	Engelsen 2010 [61]
Change in physical activity	Jorde 2010 [2]
Change in season from winter/spring to summer/autumn	Hypponen 2007, Kroll 2015 [46,47]
*For reductions in 25(OH)D concentrations*	
Decline with age due to reduced production from solar UVB	Chalcraft 2020 [62]
Change in diet with reduced meat and fish consumption	Crowe 2011 [63]
Increase in body mass	Jorde 2011 [64]
Increase in use of sunscreen/sunblock, clothing when in sunlight	Maghfour 2022 [65]
Increased use of sunscreen in cosmetics	Ngoc 2019 [66]
Moving into residential care	Okan 2020 [67]

25(OH)D, 25-hydroxyvitamin D; UVB, ultraviolet-B.

**Table 8:** Mechanisms by which vitamin D can reduce risk of stroke and MACEs.

Mechanism	Ref.
Antifibrotic, antihypertrophic signaling	Latic 2020 [80]
Anti-inflammatory, antioxidant effects	Della Nera 2023 [81]
Atherosclerosis progression reduction	Marek 2022 [82]
Reduces arterial stiffness and narrowing of the vessel lumen due to activation of the renin–angiotensin–aldosterone system	Marek 2022 [82]
Cellular effects through effects on genes (cell cycle, proliferation, apoptosis, and angiogenesis)	Marek 2022 [82]
Endothelial function maintenance	Kim 2020 [83]
Insulin resistance risk reduction	Contreras-Bolivar 2021 [84]
Lipid metabolism regulation	Surdu 2021 [85]
MMP-2 and MMP-9 activity reduced, which reduces acute arterial event risk	Timms 2002, Li 2020 [72,73]
Neuroprotective growth factor promotion	Yarlagadda 2020 [86]
Reduced risk of plaque instability and acute arterial events	Legarth 2019 [87]
Reduction in blood pressure through vasodilation	Yarlagadda 2020 [86]
Reduction in arterial pressure through effects on endothelial and muscle cells	de la Guia-Galipienso 2021 [88]
Type 2 diabetes mellitus risk reduction	Dawson-Hughes 2020 [89]

MACE, major cardiovascular event; MMP, matrix metalloproteinase.

**Table 9:** Evaluation of findings relating serum 25(OH)D concentration to the risk of stroke and of major cardiovascular disease events using Hill’s criteria for causality in a biological system [99].

Criterion	Strength of Finding	Ref.
Strength of association	“Strong”, as suggested by Figure 1 and Figure 2.	
Consistency	“Strong”, i.e., results from various European countries as well as the United States and Hong Kong were in general agreement with each other.	
Temporality	“Strong”, as all the prospective cohort studies agreed.	
Biological gradient	“Strong”, as the inverse serum 25(OH)D concentration–risk relationship is well known across the literature on human health.	Shi 2020 [42]
Plausibility	“Strong”, as many mechanisms are now well understood (see Table 7 and preceding text).	
Coherence	“Strong” because vitamin D has many mechanisms for maintaining good health, including affecting gene expression and, for example, downregulating adverse effects on immune and inflammatory processes in vivo.	Shirvani 2019 [71]
Experiment	“Weak”, as RCTs have not found that vitamin D supplementation reduces risk of stroke or of CVD events, probably due to not conducting RCTs of appropriate design (see the Discussion Section).	Pilz 2022; Grant 2022; Barbarawi 2019 [95,96,105]
Analogy	Similar findings are seen for Alzheimer’s disease, dementia, and cognitive decline.	Grant 2024 [8]
Confounding factors	A possible confounding factor is the release of nitric oxide from subcutaneous nitrate stores through the action of UV irradiation, though strong evidence for that effect is lacking.	Quan 2023 [106]

25(OH)D, 25-hydroxyvitamin D; CVD, cardiovascular disease; RCT, randomized controlled trial.

## Data Availability

The original contributions presented in the study are included in the article or in the references provided. Further inquiries can be directed to the corresponding author.
